# Multimodal integration of pathomics and single-cell transcriptomics reveals CDC20 as a link between prostate cancer recurrence and benzo[a]pyrene-responsive phenotypes

**DOI:** 10.3389/fcell.2026.1965589

**Published:** 2026-09-10

**Authors:** Xuchao Dai, Wei Gu, Bo Yu, Tao Li, Jianguo Zhu

**Affiliations:** 1 Department of Urology, The Affiliated Hospital of Guizhou Medical University, Guiyang, China; 2 Department of Urology, The Second Affiliated Hospital of Zunyi Medical University, Zunyi, China; 3 Department of Urology, Guizhou Provincial People’s Hospital, Guiyang, China

**Keywords:** benzo[a]pyrene, cdc20, network toxicology, pathomics, prostate cancer, recurrence, single-cell transcriptomics

## Abstract

**Introduction:**

Prostate cancer recurrence reflects molecular and histological heterogeneity, yet the cellular states harboring recurrence-associated signals and their potential environmental modifiers remain incompletely understood.

**Methods:**

We integrated single-cell RNA sequencing, bulk transcriptomes with recurrence annotation, quantitative histopathology, cross-cohort survival modeling, reverse network toxicology, molecular simulation, and cellular perturbation experiments.

**Results:**

Analysis of 36,025 cells from GSE141445 identified 14,464 malignant luminal epithelial cells. Scissor identified a recurrence-associated transcriptional state enriched for adhesion, migration, angiogenesis, and proliferation programs. H&E-derived features from 304 paired TCGA-PRAD cases captured variation in this transcriptional program and supported internal recurrence risk stratification. Cross-cohort modeling prioritized CDC20, ENSA, and PTTG1. Reverse toxicology further prioritized benzo[a]pyrene (BaP), and CDC20 showed the most favorable predicted BaP docking score. In PC-3 and DU145 cells, 10 nM BaP increased CDC20 expression, whereas CDC20 silencing attenuated BaP-associated proliferation, colony formation, wound closure, and migration.

**Discussion:**

These findings identify CDC20 as a recurrence-associated molecular node involved in BaP-responsive malignant biological phenotypes and provide a phenotype-anchored framework linking recurrence biology with environmental exposure-related tumor behavior.

## Highlights

- Scissor maps recurrence-associated signals to malignant luminal epithelial cells.
- H&E pathomics captures recurrence-associated transcriptional variation.
- Cross-cohort modeling prioritizes CDC20 as a robust risk-associated candidate gene.
- CDC20 silencing attenuates BaP-associated malignant biological phenotypes *in vitro*.

## 1 Introduction

Prostate cancer is one of the most common malignancies in men and remains a major source of global cancer-related disease burden (Bray et al., 2024). Advances in radical prostatectomy, radiotherapy, androgen receptor-directed therapy, and imaging techniques have extended disease control for many patients (Chakrabarti et al., 2025); however, a substantial proportion still experience biochemical recurrence, local progression, or metastasis. Traditional variables such as prostate-specific antigen, pathological stage, Gleason grade, lymph node status, and surgical margin status provide important reference value but cannot fully explain the marked differences in post-treatment outcomes. Therefore, a more refined understanding of recurrence biology requires models that link patient-level outcomes to the tumor cell states and tissue phenotypes that produce them.

Bulk transcriptomes reflect the average molecular state of tumors and cannot accurately attribute clinical signals to specific cell populations. Single-cell RNA sequencing addresses this limitation by resolving malignant epithelial and microenvironmental cell states, while Scissor associates bulk phenotypes with individual cells based on expression similarity and network-constrained regression (Sun et al., 2022). Because cell–phenotype assignments are influenced by model regularization and data structure, such labels are more appropriately interpreted as phenotype-associated transcriptional states rather than fixed cell lineages (Gan et al., 2024). Digital pathology provides another complementary scale of evidence. Recent studies have shown that routine hematoxylin and eosin (H&E) images contain features associated with biochemical recurrence and risk stratification in prostate cancer (Pinckaers et al., 2022; Shao et al., 2024; Cao et al., 2025), and integrated frameworks combining pathomics with single-cell transcriptomics have successfully identified prognosis-associated molecular programs in other cancer types (Zhao S. et al., 2025; Li et al., 2026). Therefore, integrating these modalities holds promise for revealing whether recurrence-associated cellular programs also leave measurable imprints in tissue architecture.

Environmental exposure further increases the heterogeneity of prostate cancer. Polycyclic aromatic hydrocarbons (PAHs) originate from incomplete combustion and can enter the human body through ambient air, food, and other environmental routes (Zhao H. et al., 2025). Benzo [a]pyrene (BaP) is a widely studied PAH that can be metabolically activated by aryl hydrocarbon receptor (AhR)-regulated enzymes (including CYP1A1 and CYP1B1) to generate reactive intermediates that affect DNA integrity and cell signaling. PAH–DNA adduct burden in prostate tissue is associated with early biochemical recurrence after prostatectomy (Rybicki et al., 2008), and experimental studies have shown that BaP effects vary with dose, exposure duration, and cellular context (Hruba et al., 2011; Gao et al., 2020; Nwagbara et al., 2007). Population genetic studies suggest that BaP-responsive enhancer variants can influence prostate cancer susceptibility (Fan et al., 2024); more recently, a study by Zhang et al. using cellular, patient-derived organoid, and mouse models further linked BaP exposure to prostate cancer growth and immunosuppression (Zhang Z. et al., 2025). These observations support a phenotype-anchored research strategy: starting from recurrence biology, we further investigate which environmental chemicals may converge on the same molecular nodes.

Cell division cycle 20 (CDC20) is a coactivator of the anaphase-promoting complex/cyclosome (APC/C) and a core regulator of mitotic progression (Zhang Y. et al., 2025). In prostate cancer, high CDC20 expression is associated with adverse clinicopathological features and shorter biochemical recurrence-free survival (Mao et al., 2016). Mechanistic studies have linked CDC20 to Axin1 degradation and β-catenin activity, tumor cell plasticity, immune-related cell death, and c-MYC/PI3K–AKT signaling (Zhang et al., 2019; Wu et al., 2023; Ding et al., 2026). Recent multi-omics toxicology studies have also prioritized CDC20 in pollutant-associated prostate cancer networks (Liu et al., 2026; Zhang et al., 2026), but direct functional evidence supporting CDC20 involvement in BaP-responsive phenotypes remains limited.

This study integrates recurrence-associated single-cell analysis, H&E pathomics, multi-cohort transcriptomic modeling, reverse network toxicology, molecular simulation, and *in vitro* perturbation experiments to test whether different data layers converge on the same recurrence-associated molecular node and whether this node participates in the malignant biological responses observed after low-dose BaP exposure. The results identify CDC20 as a candidate bridge connecting recurrence-associated luminal epithelial cell programs with BaP-responsive prostate cancer phenotypes, without assuming that public clinical cohorts contain individual-level BaP exposure information or treating computational docking as evidence of direct binding.

## 2 Materials and Methods

### 2.1 Study design and data sources

The analytical workflow of this study integrated public single-cell RNA sequencing data, TCGA-PRAD whole-slide images (WSIs), paired bulk RNA sequencing profiles, and recurrence-associated clinical annotations. GSE141445 was used for single-cell analysis. TCGA-PRAD RNA sequencing data and clinical annotations were obtained through UCSC Xena/GDC resources, and diagnostic H&E WSIs were obtained from the GDC Data Portal. DKFZ2018 data were obtained through cBioPortal, and GSE21032 and GSE70768 were obtained from the Gene Expression Omnibus.

For WSI analysis, cases were excluded if diagnostic slides lacked paired transcriptomic data or time-to-event information, were non-tumor or duplicate specimens, contained insufficient tissue, or had severe blurring, folding, pen marks, or staining artifacts. After quality control, 304 TCGA-PRAD cases were retained. For transcriptomic survival analysis, recurrence-associated survival endpoints annotated in the original studies or databases were used for each cohort. Because the naming and definition of endpoints varied across cohorts, including disease-free survival (DFS), progression-related survival endpoints, and biochemical recurrence, these endpoints were collectively referred to as recurrence-related outcomes for cross-cohort comparison.

### 2.2 Single-cell RNA sequencing data processing

The annotated GSE141445 Seurat object was processed using Seurat (Stuart et al., 2019). Count data were normalized using LogNormalize, highly variable genes were selected using the variance-stabilizing transformation pipeline, and principal component analysis was performed after data scaling. Sample identity was used as the grouping variable for Harmony batch integration (Korsunsky et al., 2019). A nearest-neighbor graph was constructed based on Harmony embeddings to define cell clusters and generate uniform manifold approximation and projection (UMAP) coordinates. Major cell lineages were verified using canonical markers, including EPCAM/KRT8/KRT18 for epithelial cells, CD3D/CD3E/NKG7 for T/NK cells, LYZ/CD68/C1QA for myeloid cells, PECAM1/VWF for endothelial cells, COL1A1/DCN/ACTA2 for fibroblasts or mural cells, TPSAB1/TPSB2 for mast cells, and MS4A1/CD79A for B cells (Chen et al., 2021). Subsequent phenotypic analysis focused on malignant luminal epithelial cells.

### 2.3 Mapping recurrence phenotypes using scissor

To identify single-cell subpopulations associated with the prostate cancer recurrence phenotype, the Scissor algorithm (Single-cell Identification of Subpopulations with Clinical Information) was used to integrate clinical recurrence information from TCGA-PRAD patients into single-cell transcriptomic data.

First, gene-level matching was performed between the single-cell RNA sequencing data and TCGA-PRAD bulk RNA-seq data, retaining only genes common to both expression matrices for subsequent analysis. Patients with both gene expression data and clinical recurrence status information were included, and patient recurrence status was used as the clinical phenotype input.

Scissor identifies cell subpopulations significantly associated with patient recurrence risk by building an association model between single-cell expression profiles and bulk RNA-seq clinical phenotypes. Based on the cell correlation coefficients calculated by the Scissor model, single cells were classified into different categories: cells with positive correlation coefficients were defined as Scissor + cells, representing transcriptional features associated with the tumor recurrence phenotype; cells with negative correlation coefficients were defined as Scissor− cells, representing transcriptional features associated with lower recurrence risk; and cells showing no significant association were defined as background cells. Subsequently, the Scissor classification results and corresponding correlation coefficients were added to the meta. data of the Seurat object, and the distribution characteristics of Scissor + cells across different cell populations were further analyzed in combination with cell type annotation results. In subsequent analyses, the Scissor + cell population was the focus, with differential expression analysis to screen for recurrence-associated genes, followed by functional enrichment analysis and prognostic model construction.

Differential expression between Scissor+ and Scissor− cells was assessed using Seurat FindMarkers. Genes meeting adjusted P < 0.05 and |average log2 fold change| ≥ 0.25 were included in subsequent analysis. Gene Ontology (GO) and Kyoto Encyclopedia of Genes and Genomes (KEGG) enrichment analyses were performed with Benjamini–Hochberg correction. GO terms with false-discovery rate (FDR) < 0.05 were considered statistically significant. KEGG results reaching only nominal P < 0.05 were considered exploratory and were not described as FDR-significant.

### 2.4 WSI preprocessing and pathomics feature extraction

Tissue regions were first separated from slide background, and non-overlapping 512 × 512-pixel tiles were generated at ×20 magnification. Macenko normalization was used to reduce staining variation between slides (Macenko et al., 2009). Tiles with tissue coverage <80% or visible artifacts were excluded. Two complementary feature types were extracted. First, a 2,048-dimensional vector was generated from each tile resized to 224 × 224 pixels using an ImageNet-pretrained ResNet-50 (PyTorch implementation); tile-level vectors were averaged to obtain patient-level deep features. Second, handcrafted feature extraction was performed using CellProfiler v4.2.8 (McQuin et al., 2018): the UnmixColors module (optical density deconvolution) was used to separate hematoxylin and eosin channels, nuclei were segmented from the hematoxylin channel using the IdentifyPrimaryObjects module (size range: 5–50 pixels), and multi-scale descriptors covering texture, intensity, pixel distribution, and morphological features were extracted to characterize cytological and histological variation. Patient-level features were standardized using Z-score. CellProfiler features were used to characterize morphological networks, while the final WSI survival model was built on ResNet-50 features.

### 2.5 Association of pathomics features with recurrence-associated programs

In the paired TCGA-PRAD cohort, a pathology-omics feature matrix was constructed integrating ResNet-50 deep features and CellProfiler handcrafted features. Gene set variation analysis (GSVA) (Hänzelmann et al., 2013) was performed on paired TCGA-PRAD bulk expression profiles using the Scissor + marker gene set. To prevent information leakage, only overlapping samples with both WSI and transcriptomic data were included. Two-sided Spearman correlation analysis was used to test associations between GSVA scores and individual pathomics features. Benjamini–Hochberg correction was applied across all features, with FDR <0.05 defining significant associations. Features passing the significance threshold constituted the “correlation candidate set.” Genes associated with significant image features formed the candidate space for subsequent cross-cohort modeling.

### 2.6 Internal recurrence risk modeling from WSI

Using a fixed random seed, 304 eligible TCGA-PRAD cases were randomly divided into an internal training set (n = 243) and an internal test set (n = 61) at an 8:2 ratio. Feature selection and model fitting were performed only in the training set. Univariate Cox analysis was first used for preliminary screening, followed by evaluation of 101 machine learning algorithm combinations within the Mime framework (Liu et al., 2024). Tree-based and Cox-type models showed the highest stability. The combination of forward stepwise Cox regression and gradient boosting machine (StepCox [forward] + GBM) was ultimately selected to build the WSI model, achieving optimal discrimination with the lowest overfitting in cross-validation. Patients were grouped by the median training set risk score. Model discrimination was assessed using the concordance index and time-dependent receiver operating characteristic curves, with cross-validation in the training set and independent evaluation in the test set. Kaplan–Meier curves and log-rank tests were used to assess survival differences. In multivariable Cox models, clinical covariates were included together with the risk score. This data split constitutes only an internal hold-out evaluation and is not described as external WSI validation.

### 2.7 Cross-cohort transcriptomic survival modeling

One hundred one combinations derived from 10 survival learning algorithms were used to evaluate pathology-associated candidate genes. TCGA-PRAD was used for model fitting, and DKFZ 2018, GSE21032, and GSE70768 were used to assess cross-study reproducibility. Models were ranked by their average concordance index across the four cohorts, and SuperPC and RSF + SuperPC were included in subsequent analysis. Because performance across all cohorts participated in model ranking, these analyses are described as cross-cohort evaluation rather than fully independent external validation not involved in model selection. RSF + SuperPC scores were used for risk stratification. Kaplan-Meier analysis, hazard ratios with 95% confidence intervals, and 1-, 3-, and 5-year time-dependent AUCs were reported separately for each cohort. Genes shared by the two best-performing models were further evaluated individually, and those showing directionally consistent adverse associations across cohorts were prioritized for subsequent environmental factor retrieval.

### 2.8 Reverse network toxicology and protein expression context

The Comparative Toxicogenomics Database (CTD) (Davis et al., 2025) was queried using CDC20, ENSA, and PTTG1 as search terms. Only chemical–gene records meeting the following criteria were retained: involving a single chemical, species *Homo sapiens*, increased target gene mRNA expression, and original evidence records classified as marker/mechanism with corresponding citations. Records involving multi-chemical co-exposure, indirect evidence, uncertain species attribution, or insufficient experimental source information were excluded. Before prioritization, pharmacological or experimental interventions were distinguished from environmental pollutants. Representative immunohistochemistry images of CDC20, ENSA, and PTTG1 were obtained from the Human Protein Atlas (HPA) (Uhlen et al., 2015) and were used solely to display protein expression context, not as an independently quantified tissue cohort.

### 2.9 Molecular docking and exploratory molecular dynamics

Ligand structures were obtained from PubChem, and target protein structures were obtained from the AlphaFold Protein Structure Database (Varadi et al., 2022). Proteins and ligands were preprocessed using AutoDockTools 1.5.7, and predicted binding conformations and scores were compared by molecular docking (Morris et al., 2009). Docking results are reported as scoring function estimates in kcal/mol; in the selected model, more negative values indicate more favorable predicted conformations, but these values are not equivalent to measured binding free energies and cannot serve as evidence of target binding.

A 100 ns molecular dynamics simulation of the top-ranked CDC20–BaP docking conformation was performed using GROMACS 2023.2 (Abraham et al., 2015). CDC20 was parameterized using CHARMM36; ligand topology was generated by Sobtop according to the parameters used in the original analysis. The system was solvated with the modified TIP3P water model, electrically neutralized, and adjusted to 0.15 M NaCl. After steepest descent energy minimization, 100 ps NVT equilibration and 100 ps NPT equilibration were performed sequentially, followed by a 100 ns production simulation with a 2 fs time step. Calculated metrics included root-mean-square deviation (RMSD), root-mean-square fluctuation, radius of gyration, solvent-accessible surface area, and principal component analysis-based free energy landscape. Given that the protein and ligand parameterization schemes were not derived from a unified force field workflow and that experimental binding assays were lacking, this simulation is considered only exploratory structural supporting evidence.

### 2.10 Cell culture and BaP exposure

PC-3, DU145, and RWPE-1 cells were purchased from the Cell Bank of the Chinese Academy of Sciences and cultured according to the supplier’s recommended conditions at 37 °C with 5% CO2. BaP (Sigma-Aldrich, B1760; purity ≥96%) was dissolved in dimethyl sulfoxide (DMSO), aliquoted, protected from light, and stored at the temperature recorded in the laboratory protocol. The final DMSO concentration was 0.1% (v/v) in all exposure and solvent control groups. Based on preliminary cell viability assessment and previous low-dose studies, 10 nM BaP treatment for 24 h was selected as the working exposure condition. Cells were subsequently collected for expression analysis or used in corresponding functional experiments.

### 2.11 Lentivirus-mediated CDC20 silencing

Three non-overlapping CDC20 short hairpin RNAs (sh-CDC20-1, sh-CDC20-2, and sh-CDC20-3) and a non-targeting control (sh-NC) were cloned into the pLKO.1-puro vector. Lentiviral particles were produced in HEK293T cells, and supernatants collected at 48 and 72 h post-transfection were filtered and pooled. PC-3 and DU145 cells were infected at a multiplicity of infection of 5. Medium was changed after 24 h, and cells were selected with 1 μg/mL puromycin for 3 days. Knockdown efficiency was screened by RT-qPCR and immunoblotting. The final figure legends must indicate the sequence used for each downstream experimental panel; all sequences are listed in Supplementary Table S2.

### 2.12 RT-qPCR and immunoblotting

Total RNA was extracted using TRIzol. Samples with OD260/280 ≥1.9 were reverse-transcribed using PrimeScript RT Master Mix. Quantitative PCR was performed using iTaq Universal SYBR Green Supermix on a QuantStudio instrument with the following program: 95 °C for 3 min, followed by 40 cycles of 95 °C for 10 s, 59 °C for 30 s, and 72 °C for 30 s. GAPDH was used as the reference gene, and relative expression was calculated using the 2^−ΔΔCT^ method. Primer sequences are listed in Supplementary Table S1.

For immunoblotting, cells were lysed with RIPA buffer supplemented with protease inhibitors, and protein concentration was determined using the BCA assay. Equal amounts of protein were separated on 10% SDS-polyacrylamide gels and transferred to PVDF membranes. Membranes were blocked with 5% non-fat milk for 1 h, then incubated overnight at 4 °C with anti-CDC20 antibody (1:2,000; Proteintech, 10252-1-AP) and a laboratory-validated GAPDH loading control antibody. After TBST washes, membranes were incubated with species-matched secondary antibodies for 2 h.

### 2.13 Cell proliferation, colony formation, wound healing, and transwell migration assays

For the EdU incorporation assay, 3 × 10^3^ cells per well were seeded in 24-well plates. After the indicated treatments, cells were incubated with 10 μM EdU for 2 h, fixed, and processed according to the instructions of the E-Click EdU Cell Proliferation Imaging Assay Kit (Elabscience, Wuhan, China; cat. no. E-CK-A377), followed by counterstaining with the kit-provided DAPI Reagent (Elabscience; cat. no. E-CK-A163; working concentration 1 μg/mL). The EdU-positive proportion was calculated using the total number of DAPI-positive nuclei as the denominator. Fluorescence images were acquired using an ECLIPSE Ti-S inverted fluorescence microscope (Nikon, Tokyo, Japan) equipped with a ×20 objective. EdU signals were detected in the Elab Fluor® 594/TRITC channel (Ex/Em 590/617 nm) and DAPI signals in the DAPI channel (Ex/Em 350/470 nm). Exposure times were 300 m for EdU and 10 m for DAPI, and identical imaging settings were maintained across all experimental groups.

For the colony formation assay, cells were seeded in 6-well plates, allowed to adhere, treated with 10 nM BaP or solvent control, and cultured for 14 days. Colonies were fixed with 4% paraformaldehyde, stained with 0.1% crystal violet, and quantified according to the pre-specified analysis unit.

For the wound healing assay, a sterile 200 μL pipette tip was used to scratch the confluent cell monolayer, which was then washed and cultured in low-serum medium. Images were acquired at the same marked positions at 0 and 48 h. Wound closure rate was calculated as (area_0_ h − area_48_ h)/area_0_ h × 100%. For the Transwell assay, cells suspended in serum-free medium were added to the upper chamber of 8 μm pore inserts, with medium containing 10% fetal bovine serum as the chemoattractant, and migrated for 24 h. Cells on the lower surface were fixed, stained with crystal violet, and imaged and counted in 5 random fields. Because laboratory records did not indicate the use of extracellular matrix coating, this endpoint is referred to as migration rather than invasion.

### 2.14 Statistical analysis

Continuous cell experiment data are presented as mean ± standard deviation. Replicate well data within each independent experiment were averaged first and were not treated as independent biological samples. For two-group comparisons, two-sided unpaired Student's t-test or Welch’s test was used when distribution assumptions were met; otherwise, the Mann–Whitney U test was used. One-way analysis of variance with multiplicity-corrected *post hoc* tests, or corresponding non-parametric methods, was used to compare the four existing conditions (DMSO, BaP, BaP + sh-NC, and BaP + sh-CDC20). Two-sided Wilcoxon tests were used for single-cell pairwise comparisons. Benjamini–Hochberg correction was applied for differential expression, enrichment, and high-dimensional correlation analyses. Time-to-event analyses report hazard ratios, 95% confidence intervals, and log-rank P values. All analyses were performed in R, Python, and GraphPad Prism 9. Unless an FDR threshold is otherwise specified, two-sided P < 0.05 was considered statistically significant.

## 3 Results

### 3.1 Malignant luminal cell population dominates the single-cell epithelial atlas

**FIGURE 1:**
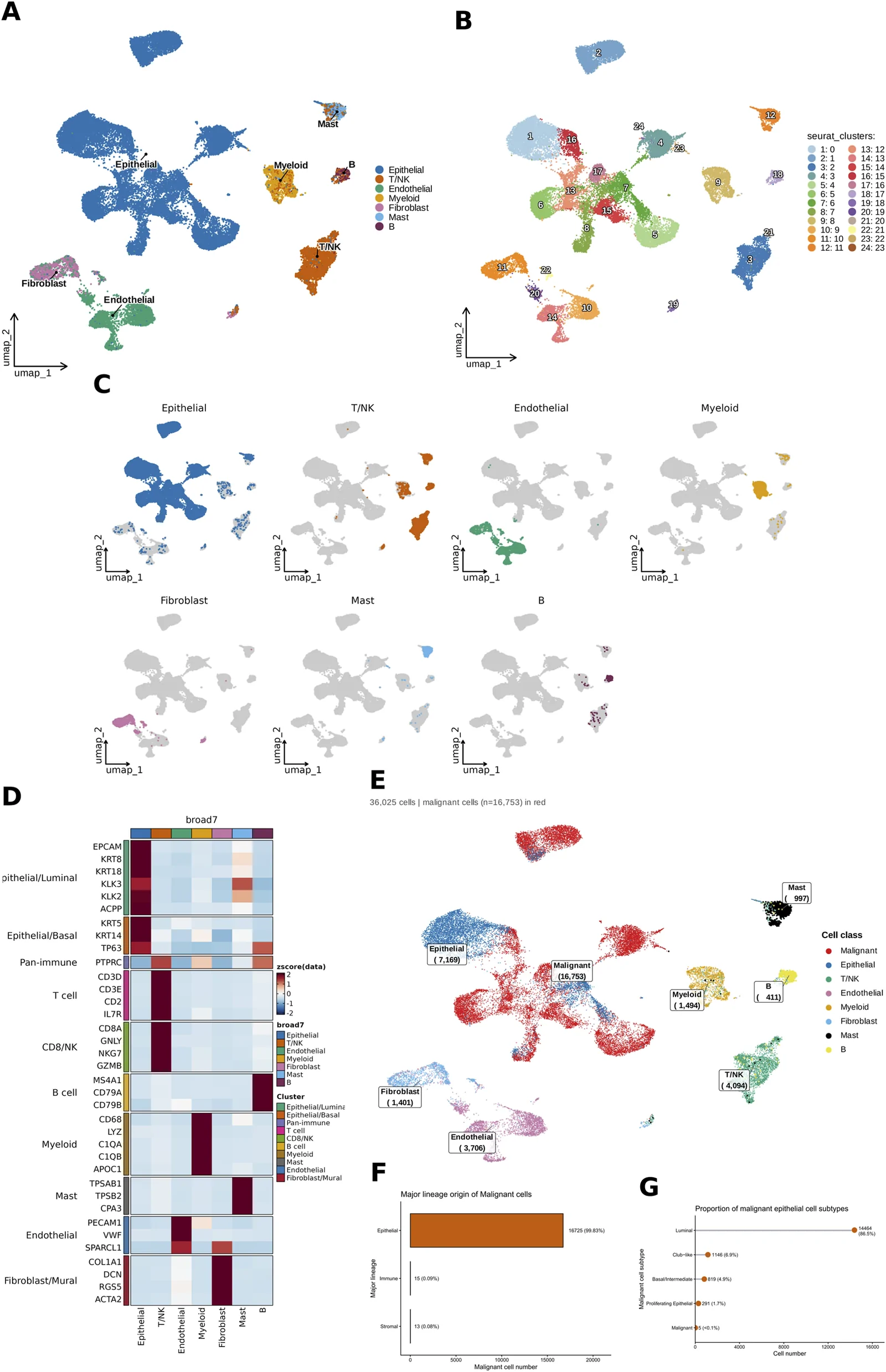
Single-cell atlas of prostate cancer and the malignant luminal cell compartment. **(A)** UMAP plot of seven major cell lineages. **(B)** Unsupervised clustering results. **(C)** UMAP facet plots for each cell lineage. **(D)** Heatmap of canonical marker genes. **(E)** Distribution and number of malignant and non-malignant cells. **(F)** Lineage origin of malignant cells. **(G)** Composition of malignant epithelial cell subtypes.

The integrated GSE141445 atlas contained 36,025 cells assignable to 7 major lineages: epithelial cells, T/NK cells, endothelial cells, myeloid cells, fibroblasts, mast cells, and B cells (Figures 1A–D). Unsupervised analysis identified 24 transcriptionally distinct cell clusters. Marker expression was consistent with corresponding lineage identities, including epithelial keratins, lymphocyte markers, endothelial markers PECAM1/VWF, myeloid markers LYZ/C1QA, and fibroblast or vascular wall cell markers COL1A1/DCN/ACTA2.

Among the 36,025 cells, 16,753 were annotated as malignant cells (Figure 1E). Almost all malignant cells originated from the epithelial lineage (16,725; 99.83%), with only a small number assigned to immune or stromal lineages (Figure 1F). Within the malignant epithelial cell population, luminal cells predominated (14,464; 86.5%), followed by club-like cells (1,146; 6.9%), basal/intermediate cells (819; 4.9%), and proliferative epithelial cells (291; 1.7%) (Figure 1G). Therefore, subsequent phenotypic mapping analyses focused on the malignant luminal cell population.

### 3.2 Recurrence-associated scissor states exhibit distinct motility and proliferation programs

**FIGURE 2:**
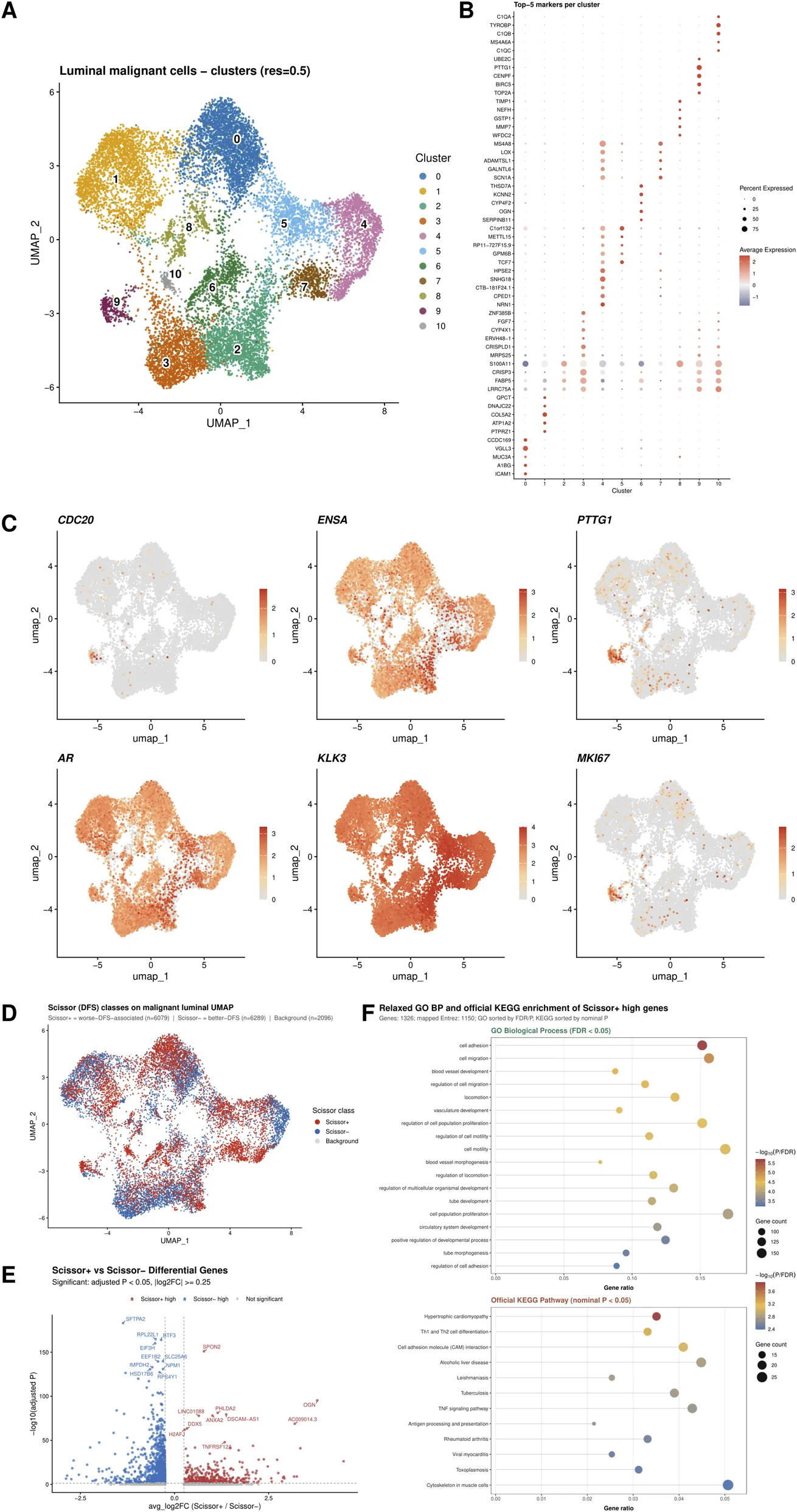
Recurrence-associated transcriptional states in malignant luminal cells. **(A)** UMAP plot of 11 luminal cell clusters. **(B)** Major marker genes for each cell cluster. **(C)** Feature plots for CDC20, ENSA, PTTG1, AR, KLK3, and MKI67. **(D)** Distribution of Scissor+, Scissor−, and background coefficients. **(E)** Differential expression volcano plot. **(F)** GO enrichment results with FDR <0.05, and exploratory KEGG enrichment results with nominal P < 0.05.

The 14,464 malignant luminal cells could be divided into 11 cell clusters with distinct marker profiles (Figures 2A,B). AR and KLK3 were broadly expressed, while CDC20, PTTG1, and MKI67 were mainly restricted to regions with more pronounced proliferative features in the UMAP (Figure 2C). Scissor integrated 473 TCGA-PRAD samples with recurrence annotation into the single-cell expression matrix based on 16,412 shared genes. The resulting positive and negative coefficients mapped oppositely directed recurrence associations across the luminal cell atlas (Figure 2D).

Differential expression analysis showed that the two states were clearly separated at the molecular level (Figure 2E). Genes with elevated expression in Scissor + cells included SPON2, OGN, PHLDA2, DSCAM-AS1, ANXA2, LINC01088, and AC009014.3. GO analysis showed that the Scissor + program was associated with cell adhesion, migration, motility, angiogenesis, regulation of cell population proliferation, and cell motility (FDR <0.05) (Figure 2F). KEGG categories included cell adhesion molecules, TNF signaling pathway, antigen processing and presentation, and cytoskeleton-related programs; because these KEGG results reached only the nominal significance threshold and not the FDR-corrected threshold, they are considered hypothesis-generating only.

### 3.3 H&E-derived features capture recurrence-associated molecular programs

**FIGURE 3:**
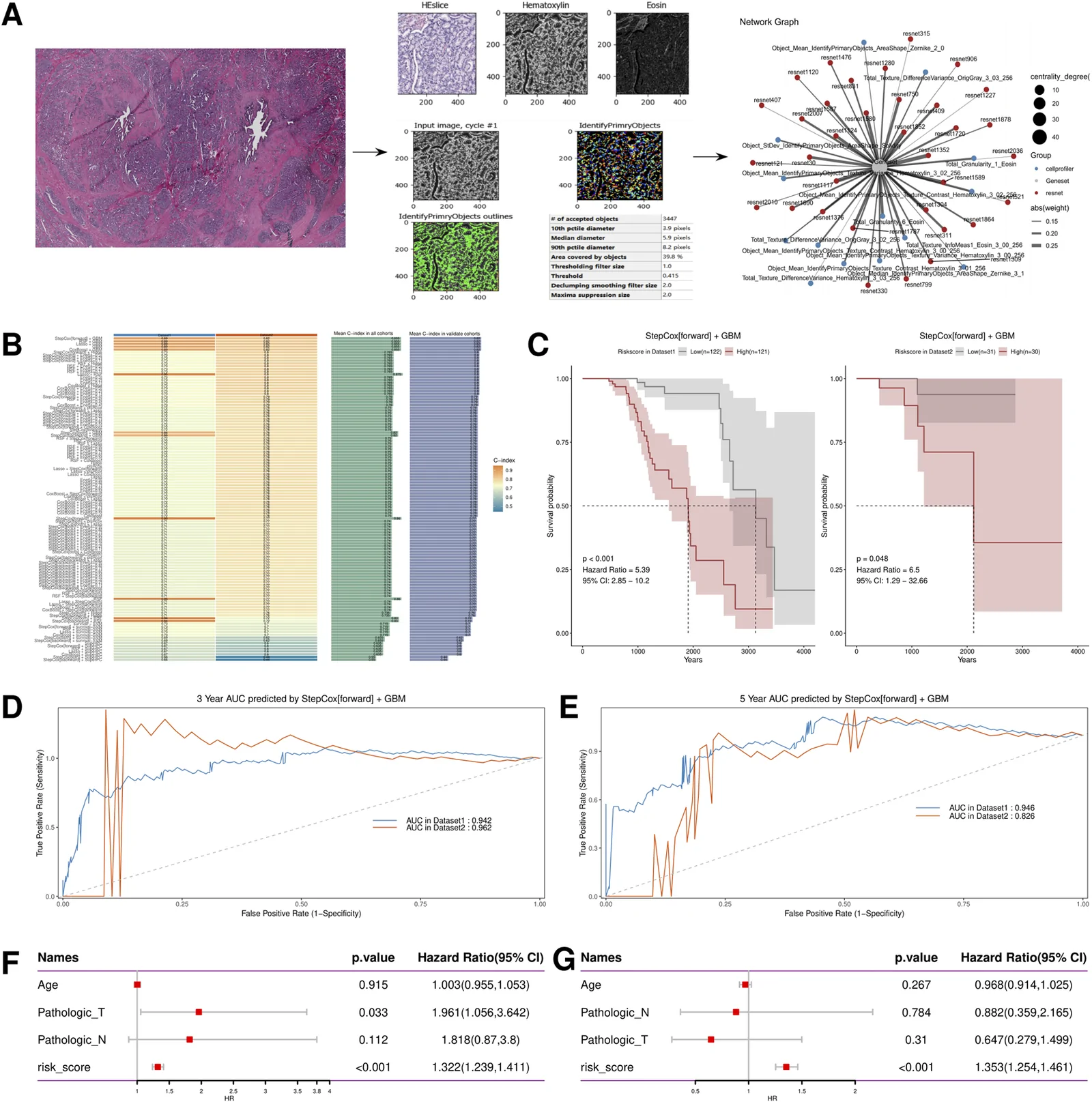
Internal development and evaluation of the H&E pathomics recurrence risk model. **(A)** WSI preprocessing, stain separation, object identification, and feature extraction workflow. **(B)** Concordance comparison of different algorithm combinations. **(C)** Kaplan–Meier curves for the internal training and test sets. **(D,E)** 3-year and 5-year time-dependent ROC curves. **(F,G)** Univariate and multivariable Cox analyses.

We next evaluated whether the Scissor + program could be reflected in routine histological images. The WSI workflow sequentially integrated tissue detection, stain separation, nuclear and texture descriptors, and ResNet-50 deep features, and correlated patient-level morphological features with bulk GSVA scores (Figure 3A). After multiple testing correction, multiple deep features and handcrafted features were associated with the recurrence-associated program, supporting a measurable correspondence between tissue architecture and this molecular state.

Among all tested combinations, the StepCox [forward] + GBM WSI model showed the strongest internal discrimination, with C-indices of 0.89 and 0.82 in the training and held-out test sets, respectively (Figure 3B). Using the median risk score as the threshold, the high-risk group in the training set had shorter recurrence-related time intervals (hazard ratio [HR] 5.39, 95% confidence interval [CI] 2.85–10.20; P < 0.001); the internal test set (n = 61) showed results in the same direction (HR 6.50, 95% CI 1.29–32.66; P = 0.048) (Figure 3C). Although the risk stratification direction was consistent, the small test set sample size and wide confidence intervals indicate limited estimation precision. The reported 3-year AUCs were 0.942 and 0.962 for the training and test sets, respectively, and the 5-year AUCs were 0.946 and 0.826 (Figures 3D,E). In multivariable analysis including age, pathological T stage, and pathological N stage, the pathomics risk score remained associated with outcome (HR 1.353, 95% CI 1.254–1.461; P < 0.001) (Figures 3F,G). These results support the internal performance of the model, but evaluation in an independent WSI cohort is still needed.

### 3.4 Cross-cohort modeling converges on CDC20, ENSA, and PTTG1

**FIGURE 4:**
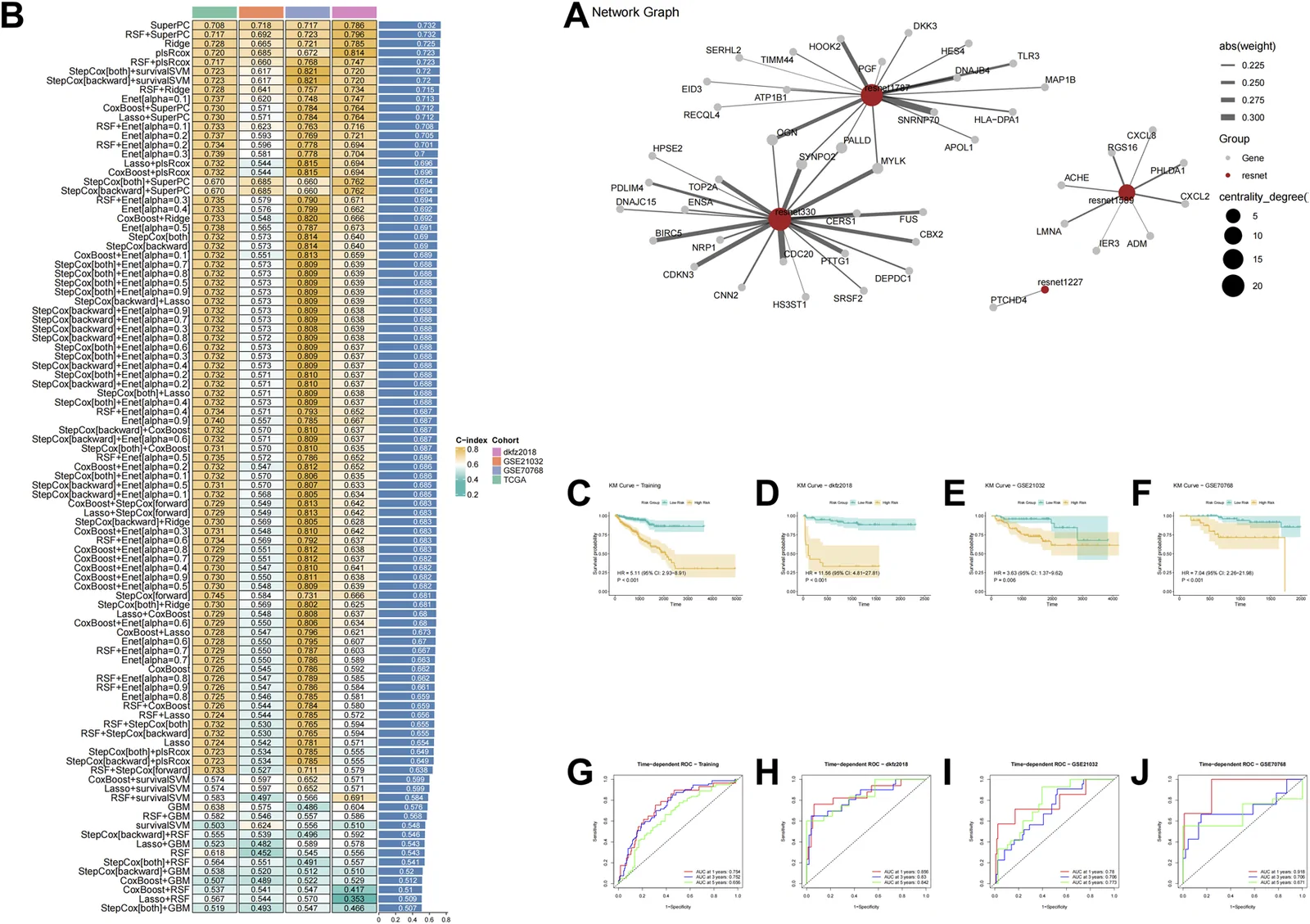
Cross-cohort modeling of pathology feature-associated genes. **(A)** Image feature–gene network. **(B)** C-index comparison of 101 algorithm combinations across four cohorts. **(C–F)** Kaplan–Meier curves for the TCGA-PRAD, DKFZ 2018, GSE21032, and GSE70768 cohorts. **(G–J)** Time-dependent ROC curves in the corresponding cohorts.

The pathology–transcriptome network showed that resnet1787, resnet1589, resnet1227, and resnet330 were the major image feature nodes connected to candidate genes (Figure 4A). Among the 101 survival learning combinations, SuperPC and RSF + SuperPC had the highest average concordance indices across TCGA-PRAD, DKFZ 2018, GSE21032, and GSE70768, both at 0.732 (Figure 4B). The intersection of the two models yielded 12 candidate genes: FUS, CDKN3, SERHL2, PTTG1, CDC20, SNRNP70, DEPDC1, CBX2, ENSA, TIMM44, HS3ST1, and HOOK2.

The RSF + SuperPC score distinguished higher-risk from lower-risk groups in TCGA-PRAD (HR 5.11, 95% CI 2.93–8.91; P < 0.001), DKFZ 2018 (HR 11.56, 95% CI 4.81–27.81; P < 0.001), GSE21032 (HR 3.63, 95% CI 1.37–9.62; P = 0.006), and GSE70768 (HR 7.04, 95% CI 2.26–21.98; P < 0.001) (Figures 4C–F). Time-dependent AUCs also showed reproducible discrimination across studies (Figures 4G–J). Subsequent gene-by-gene evaluation prioritized CDC20, ENSA, and PTTG1 because their associations with adverse recurrence-related outcomes were directionally consistent across cohorts.

### 3.5 Reverse toxicology prioritizes BaP and identifies CDC20 as the candidate protein with the best docking score

**FIGURE 5:**
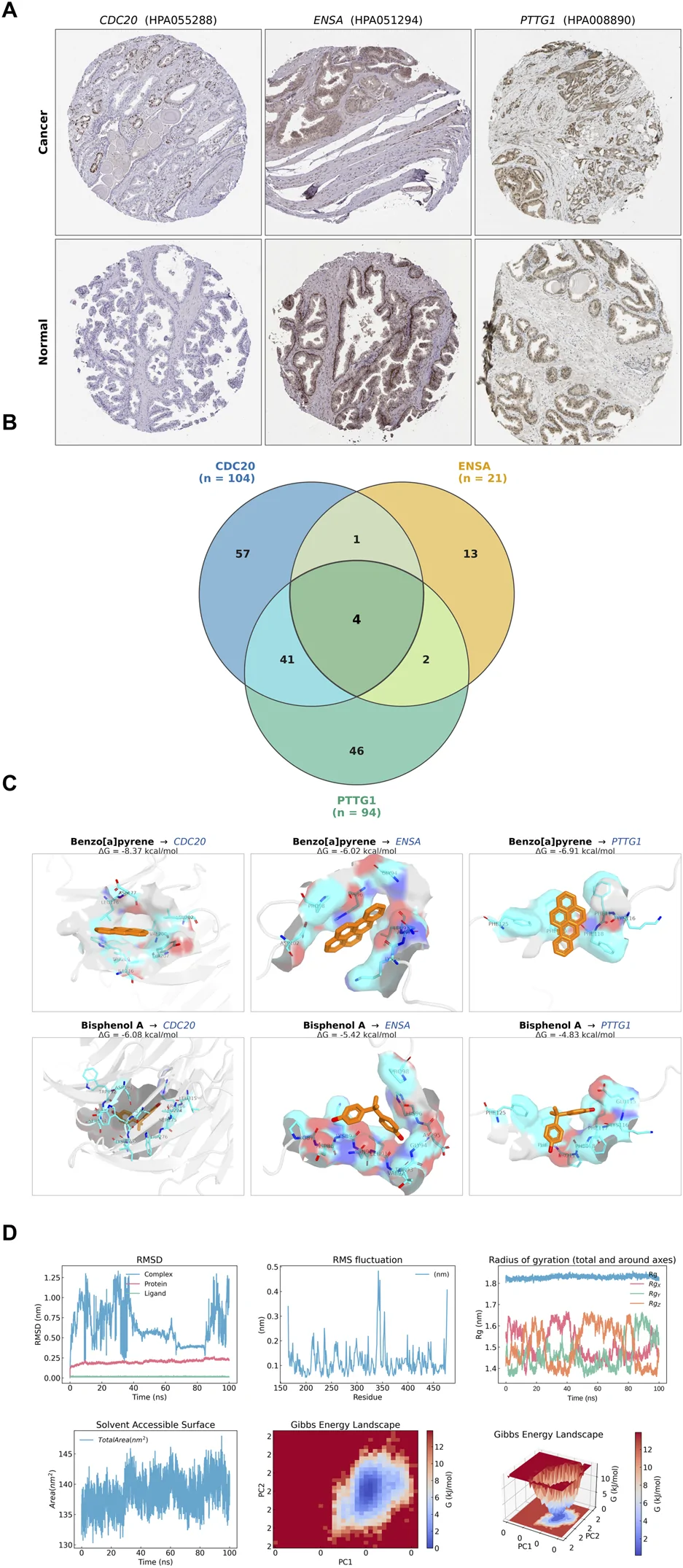
Protein expression context, reverse toxicology, molecular docking, and exploratory molecular dynamics assessment. **(A)** Representative HPA immunohistochemistry images of CDC20, ENSA, and PTTG1 in prostate cancer and non-malignant prostate tissues. **(B)** Intersection of chemicals reported to increase human CDC20, ENSA, and PTTG1 mRNA expression in CTD. **(C)** Predicted docking conformations and scores of BaP and bisphenol A with the three proteins. **(D)** RMSD, residue fluctuation (RMSF), radius of gyration, solvent-accessible surface area, and principal component analysis-based free energy landscape of the top-ranked CDC20–BaP docking conformation over the 100 ns molecular dynamics trajectory.

Representative HPA images showed staining for CDC20 and ENSA in prostate cancer tissues, while the tumor–normal tissue staining difference for PTTG1 was relatively inconspicuous (Figure 5A). CTD queries identified 4 chemicals with evidence of simultaneously increasing human CDC20, ENSA, and PTTG1 mRNA expression: pirinixic acid, cisplatin, bisphenol A, and BaP (Figure 5B). After distinguishing pharmacological or experimental interventions from environmental pollutants, bisphenol A and BaP were retained.

The docking scores of BaP with CDC20, ENSA, and PTTG1 were −8.37, −6.02, and −6.91 kcal/mol, respectively. The corresponding scores of bisphenol A with the above three targets were −6.08, −5.42, and −4.83 kcal/mol (Figure 5C). Therefore, within the scope of the selected targets and docking protocol, CDC20 obtained the most favorable predicted BaP docking conformation. Molecular dynamics trajectories supported the overall stability of the CDC20 protein fold (Figure 5D). Therefore, this study considers the molecular docking and molecular dynamics results only as structural plausibility evidence supporting subsequent functional validation. Integrating the molecular docking and molecular dynamics analyses, CDC20 represents a candidate molecular node for further investigation of BaP-related molecular mechanisms.

### 3.6 CDC20 silencing attenuates BaP-associated malignant biological phenotypes in vitro

**FIGURE 6:**
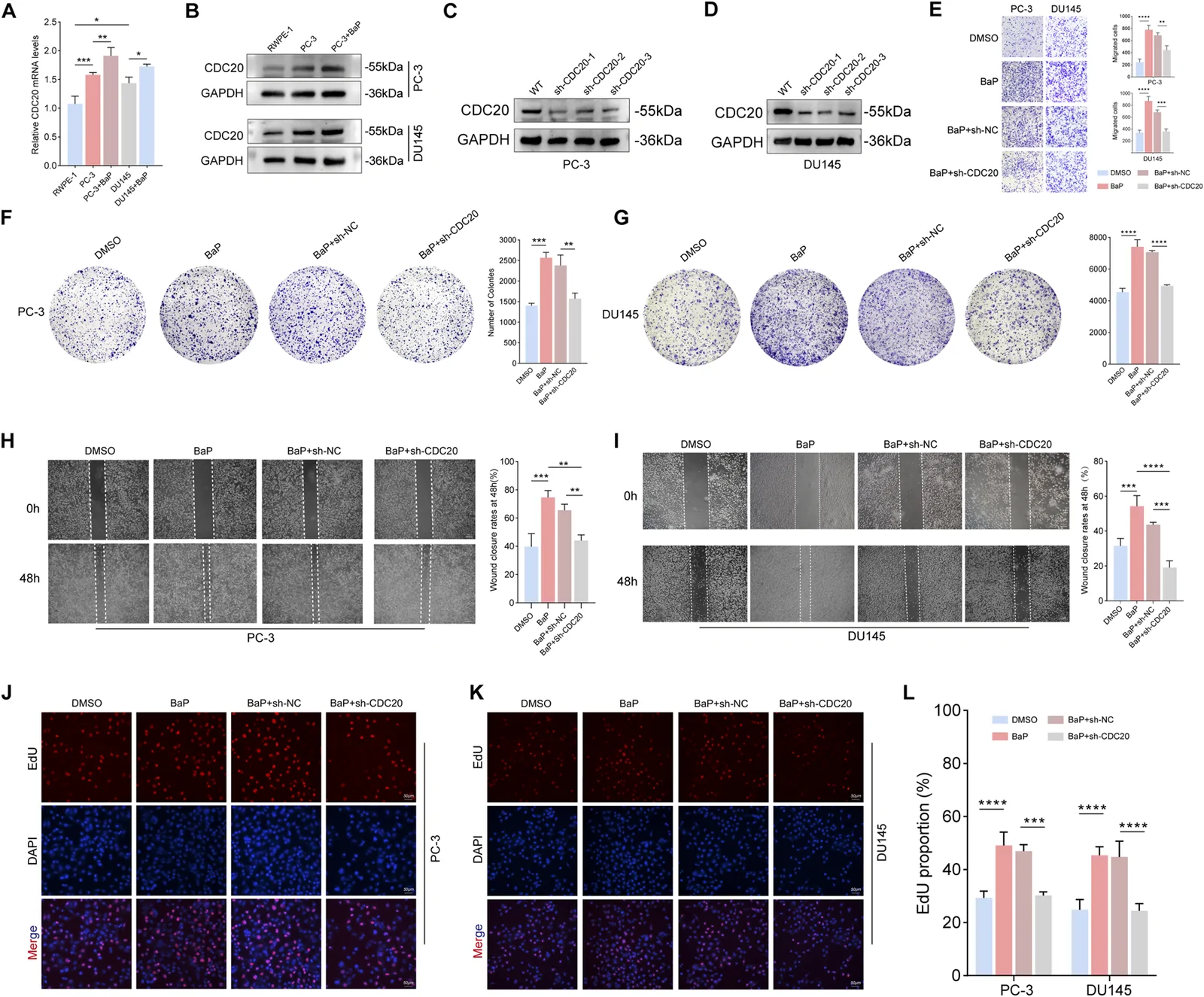
CDC20 expression and loss-of-function phenotypes under BaP exposure conditions. **(A,B)** CDC20 mRNA **(A)** and protein **(B)** expression in RWPE-1, PC-3, and DU145 cells with or without 10 nM BaP treatment for 24 h **(C,D)** Immunoblotting results screening three CDC20 shRNAs in PC-3 **(C)** and DU145 **(D)** cells. **(E)** Representative images and quantification of the Transwell migration assay. **(F,G)** Representative images and quantification of colony formation assays in PC-3 **(F)** and DU145 **(G)** cells. **(H,I)** Representative images and quantification of wound closure at 0 and 48 h in PC-3 **(H)** and DU145 **(I)** cells. **(J–L)** Representative images and quantification of EdU incorporation in PC-3 **(J)** and DU145 **(K)** cells **(L)**. Experimental groups were DMSO, BaP, BaP + sh-NC, and BaP + sh-CDC20. Sh-CDC20 refers to sh-CDC20-1 in panels E–L. Data are presented as mean ± SD from n = 3 independent biological experiments; statistical methods are described in “Materials and Methods 2.14”.

CDC20 mRNA and protein levels were higher in PC-3 and DU145 cells than in RWPE-1 cells; after 24 h of 10 nM BaP treatment, CDC20 expression was further increased in both cancer cell lines (Figures 6A,B). All three non-overlapping shRNAs reduced CDC20 protein abundance (Figures 6C,D); sh-CDC20-1 was selected for subsequent functional experiments.

Compared with DMSO, BaP exposure increased the number of migrated cells in the Transwell assay; the BaP + sh-CDC20 group had fewer migrated cells than the BaP + sh-NC group (Figure 6E). Colony formation readouts in PC-3 and DU145 cells showed similar trends (Figures 6F,G). BaP also increased the 48 h wound closure rate, while CDC20 silencing reduced the wound closure rate below that of the non-targeting control under exposure conditions (Figures 6H,I). EdU incorporation increased after BaP treatment and remained elevated under sh-NC conditions; CDC20 silencing reduced the EdU-positive proportion in both cell lines (Figures 6J–L). Overall, CDC20 silencing attenuated multiple malignant phenotypes under BaP exposure conditions.

## 4 Discussion

This study started from the clinical phenotype, a choice that allowed the analysis to trace stepwise from patient-level recurrence outcomes to the malignant luminal cell states harboring these signals, then to their histological correlates, and finally to molecular nodes that could be tested under environmental exposure conditions. Across these different levels, CDC20 was repeatedly identified: it localized to regions with proliferative features in the malignant luminal cell atlas, participated in forming pathology-associated risk programs that were consistent across cohorts, was prioritized by reverse toxicology analysis, showed increased expression after low-dose BaP exposure, and played a functional role in the full manifestation of multiple BaP-associated phenotypes. These results do not imply that BaP exposure has been measured in TCGA patients, but rather identify CDC20 as a biologically coherent candidate molecule linking recurrence-associated tumor tissue features with experimentally observed BaP responses.

Scissor analysis is central to the above interpretation. In this study, the main value of Scissor lies in bridging bulk clinical phenotypes with the single-cell transcriptional continuum (Sun et al., 2022), rather than assigning future clinical outcomes to individual cells. The Scissor + state is enriched for adhesion, migration, proliferation, and angiogenesis-related programs, indicating that recurrence associations exist within a broader malignant epithelial phenotype. This interpretation can also accommodate the wide distribution of positive and negative coefficients in the current model, as well as the known sensitivity of cell–phenotype correspondence to analytical regularization (Gan et al., 2024). Pathomics analysis further complements an intermediate scale of evidence. H&E-derived features correlated with Scissor-associated programs, and an internal model built from WSI could stratify recurrence risk. Recent prostate cancer studies have similarly shown that routine histological images can support recurrence prediction (Pinckaers et al., 2022; Shao et al., 2024; Cao et al., 2025), and pathology–single-cell integration frameworks have successfully localized prognosis-associated cell programs in melanoma and hepatocellular carcinoma (Zhao S. et al., 2025; Li et al., 2026); the added value of this study lies in linking image signals to a clearly defined malignant epithelial program, rather than treating them as purely data-driven predictors.

CDC20 has the biological basis to integrate this cross-scale signal. CDC20 is a core mitotic regulator, so its enrichment in proliferative luminal cell states is expected. However, the prostate cancer literature suggests that the significance of CDC20 is not limited to general cell cycle activity. High CDC20 expression is associated with higher Gleason score, positive surgical margins, and shorter biochemical recurrence-free survival (Mao et al., 2016). Functional studies have linked CDC20 to Axin1 degradation and β-catenin stabilization in prostate cancer stem-like cells (Zhang et al., 2019), GSDME-related pyroptosis and anti-tumor immunity (Wu et al., 2023), and c-MYC/PI3K–AKT signaling (Ding et al., 2026). The convergence of these pathways provides a reasonable explanation for CDC20’s association with both proliferation- and motility-related phenotypes in this study and makes it a suitable intervention target. From a structural perspective, the C-terminal WD40 domain of CDC20 binds APC/C substrates through a multisite degron recognition mechanism, and its substrate-binding pocket has well-defined structural features (Tian et al., 2012); this pocket is also the site of action of the small-molecule inhibitor apcin, which synergistically blocks mitotic exit with TAME, an inhibitor of APC/C–CDC20 interaction (Sackton et al., 2014). This structural background suggests that the substrate-binding region of CDC20 is accessible to small molecules, providing a structural plausibility reference for the predicted docking conformation of BaP with CDC20 in this study. It should be emphasized that the docking and molecular dynamics results in this study reflect only computational conformational preferences and cannot be equated with actual binding events; whether BaP interacts directly with CDC20 through this pocket or other sites requires experimental binding assays for validation. However, how much additional information CDC20 provides relative to the broader G2/M program remains an important open question.

The environmental exposure context of this study is supported by several complementary but not equivalent observations. PAH–DNA adducts in prostate tissue are associated with early biochemical recurrence after surgery (Rybicki et al., 2008), but such adducts do not provide BaP-specific exposure measurements. A BaP-responsive enhancer variant can alter AhR-dependent FAM227A expression and prostate cancer susceptibility (Fan et al., 2024). A recent study further found that BaP can promote prostate cancer growth and reduce CD4^+^ and CD8^+^ T cell infiltration in cellular, patient-derived organoid, and mouse models (Zhang Z. et al., 2025). At the cellular level, the direction of BaP response is highly dependent on the model used and the exposure window. LNCaP cells can show suppressed DNA replication and cell cycle programs after AhR ligand exposure (Hruba et al., 2011), while PC-3 cells can show enhanced proliferation, mutagenesis, and migration involving AhR/CYP1 and JAK2–STAT3 signaling (Gao et al., 2020). In contrast, higher micromolar concentrations of BaP or BPDE exposure can cause cytotoxicity and cell cycle arrest in DU145 cells (Nwagbara et al., 2007). Therefore, the consistent CDC20 response exhibited by two AR-negative cell lines at 10 nM defines a specific low-dose, short-duration experimental window rather than a universally applicable dose–response relationship. Whether CDC20 responds monotonically across lower and higher BaP concentrations remains unknown. Moreover, because PC-3 and DU145 are AR-negative models, whether the magnitude or direction of the CDC20 response is preserved in AR-positive prostate cancer cells requires further investigation.

Recent environmental oncology research helps clarify the academic context of this study. Recent studies have screened pollutant-associated prostate cancer targets by combining network toxicology, multi-cohort modeling, single-cell analysis, molecular docking, and limited cell validation (Liu et al., 2026; Zhang et al., 2026). BaP studies in other cancer types in EES have similarly employed multi-omics prioritization or orthogonal pathway inhibition strategies (Huang et al., 2025; Xiao et al., 2025; Fu et al., 2025). In the prostate cancer field, a recent BaP multi-omics study prioritized an RRM2-centered experimental axis while also reporting CDC20 as a prognostic and molecular docking candidate (Liu et al., 2026). Another PFAS study converged on CDC20 through a highly similar multi-omics framework and showed pollutant-associated cellular phenotypes (Zhang et al., 2026). Against this rapidly evolving research background, the unique contribution of this study is not the first identification of CDC20 in computational analysis, but rather the phenotype-anchored integration of single-cell transcriptomics, quantitative histology, and cross-cohort survival modeling, with direct testing of CDC20 loss-of-function effects under BaP exposure conditions.

Cell functional experiments further support the role of CDC20 in tumor-associated phenotypes under BaP exposure conditions. In PC-3 and DU145 cells, CDC20 silencing significantly inhibited cell proliferation, colony formation, migration, and wound closure ability compared with the non-targeting control after BaP exposure. These findings support CDC20 as a candidate molecular node associated with BaP-responsive malignant phenotypes. Because the present design did not include a corresponding CDC20-silencing condition in the absence of BaP, the current data cannot distinguish a BaP-specific dependency on CDC20 from the general biological consequences of CDC20 depletion. Combined with the molecular docking and molecular dynamics results, which provide only computational structural plausibility rather than evidence of direct binding, CDC20 may serve as a candidate molecular link between BaP exposure and malignant phenotypes of prostate cancer cells, providing an experimental basis for elucidating the potential molecular mechanisms by which BaP promotes tumor progression.

Several factors define the scope of applicability of the conclusions of this study. The clinical cohorts are all retrospective and lack patient-level BaP biomarkers; the WSI model has only undergone internal testing and has not been externally validated. *In vitro* experiments used only a single BaP concentration and exposure duration, and AR-negative cell lines. These features limit causal and clinical extrapolation but do not alter the observation that different independent data layers consistently converge on CDC20. Future research should further conduct prospective exposure cohort studies, dose–time and AhR response experiments, complete factorial intervention experiments, and direct target binding assays.

In summary, recurrence-associated single-cell analysis and quantitative histology jointly identified a luminal epithelial program with proliferative and motility features, and cross-cohort modeling positioned CDC20 at the core of this program. Reverse toxicology analysis prioritized BaP, and cell intervention experiments showed that CDC20 loss attenuates malignant biological phenotypes under low-dose BaP exposure conditions. These results support considering CDC20 as a candidate molecular bridge connecting prostate cancer recurrence biology with BaP-responsive tumor cell behavior and provide a clear basis for subsequent validation that integrates mechanistic and exposure factors.

## 5 Conclusion

After phenotype-anchored integration of single-cell transcriptomics, H&E pathomics, cross-cohort survival modeling, reverse toxicology, and cell intervention analysis, evidence from different levels converges on CDC20 in prostate cancer. CDC20 localizes to a recurrence-associated malignant luminal transcriptional program and participates in forming a pathology feature-associated risk program with cross-cohort consistency; its expression increases after 10 nM BaP exposure, and CDC20 silencing attenuates multiple BaP-associated malignant biological phenotypes in PC-3 and DU145 cells. Integrating the available evidence, CDC20 can be considered a candidate molecular node connecting prostate cancer recurrence biology with BaP-responsive tumor cell behavior and warrants further mechanistic validation.

## Data Availability

The datasets presented in this study can be found in online repositories. The names of the repository/repositories and accession numbers can be found below: [GEO under accession number GSE141445. GSE21032 and GSE70768 are available from GEO; DKFZ2018 is available through cBioPortal; TCGA-PRAD molecular, clinical, and WSI data are available through UCSC Xena and the GDC Data Portal].
