# Supplementary material for: Multimodal integration of pathomics and single-cell transcriptomics reveals CDC20 as a link between prostate cancer recurrence and benzo[a]pyrene-responsive phenotypes
Source: Front Cell Dev Biol. 2026 Sep 10;14:1965589. doi: 10.3389/fcell.2026.1965589 (PMC13601228; doi:10.3389/fcell.2026.1965589)
Supplement: Supplementary file 1 [file Table1.docx]

# Supplementary Materials

## Supplementary Table S1. RT-qPCR primer sequences

| Gene | Forward primer (5′–3′) | Reverse primer (5′–3′) | Product (bp) |
| --- | --- | --- | --- |
| CDC20 | GCACAGTTCGCGTTCGAGA | CTGGATTTGCCAGGAGTTCGG | 188 |
| GAPDH | GAAGGTCGGAGTCAACGGAT | GGTGGAATCATATTGGAACATGT | 145 |

## Supplementary Table S2. shRNA target sequences

| Name | Target sequence (5′–3′) | Vector |
| --- | --- | --- |
| sh-NC | CAACAAGATGAAGAGCACCAA | pLKO.1-puro |
| sh-CDC20-1 | ATCAGAACAGACTGAAAGTAC | pLKO.1-puro |
| sh-CDC20-2 | CAGACATTCACCCAGCATCAA | pLKO.1-puro |
| sh-CDC20-3 | TGGTGGTAATGATAACTTGGT | pLKO.1-puro |

## Supplementary Table S3. Features retained by each evaluated algorithm combination

| Algorithm combination | Retained feature |
| --- | --- |
| CoxBoost | CDC20 |
| CoxBoost | FUS |
| CoxBoost | HES4 |
| CoxBoost | PTTG1 |
| CoxBoost | SNRNP70 |
| CoxBoost+Enet[alpha=0.1] | CDC20 |
| CoxBoost+Enet[alpha=0.1] | FUS |
| CoxBoost+Enet[alpha=0.1] | HES4 |
| CoxBoost+Enet[alpha=0.1] | PTTG1 |
| CoxBoost+Enet[alpha=0.1] | SNRNP70 |
| CoxBoost+Enet[alpha=0.2] | CDC20 |
| CoxBoost+Enet[alpha=0.2] | FUS |
| CoxBoost+Enet[alpha=0.2] | HES4 |
| CoxBoost+Enet[alpha=0.2] | PTTG1 |
| CoxBoost+Enet[alpha=0.2] | SNRNP70 |
| CoxBoost+Enet[alpha=0.3] | CDC20 |
| CoxBoost+Enet[alpha=0.3] | FUS |
| CoxBoost+Enet[alpha=0.3] | HES4 |
| CoxBoost+Enet[alpha=0.3] | PTTG1 |
| CoxBoost+Enet[alpha=0.3] | SNRNP70 |
| CoxBoost+Enet[alpha=0.4] | CDC20 |
| CoxBoost+Enet[alpha=0.4] | FUS |
| CoxBoost+Enet[alpha=0.4] | HES4 |
| CoxBoost+Enet[alpha=0.4] | PTTG1 |
| CoxBoost+Enet[alpha=0.4] | SNRNP70 |
| CoxBoost+Enet[alpha=0.5] | CDC20 |
| CoxBoost+Enet[alpha=0.5] | FUS |
| CoxBoost+Enet[alpha=0.5] | HES4 |
| CoxBoost+Enet[alpha=0.5] | PTTG1 |
| CoxBoost+Enet[alpha=0.5] | SNRNP70 |
| CoxBoost+Enet[alpha=0.6] | CDC20 |
| CoxBoost+Enet[alpha=0.6] | FUS |
| CoxBoost+Enet[alpha=0.6] | HES4 |
| CoxBoost+Enet[alpha=0.6] | PTTG1 |
| CoxBoost+Enet[alpha=0.6] | SNRNP70 |
| CoxBoost+Enet[alpha=0.7] | CDC20 |
| CoxBoost+Enet[alpha=0.7] | FUS |
| CoxBoost+Enet[alpha=0.7] | HES4 |
| CoxBoost+Enet[alpha=0.7] | PTTG1 |
| CoxBoost+Enet[alpha=0.7] | SNRNP70 |
| CoxBoost+Enet[alpha=0.8] | CDC20 |
| CoxBoost+Enet[alpha=0.8] | FUS |
| CoxBoost+Enet[alpha=0.8] | HES4 |
| CoxBoost+Enet[alpha=0.8] | PTTG1 |
| CoxBoost+Enet[alpha=0.8] | SNRNP70 |
| CoxBoost+Enet[alpha=0.9] | CDC20 |
| CoxBoost+Enet[alpha=0.9] | FUS |
| CoxBoost+Enet[alpha=0.9] | HES4 |
| CoxBoost+Enet[alpha=0.9] | PTTG1 |
| CoxBoost+Enet[alpha=0.9] | SNRNP70 |
| CoxBoost+GBM | CDC20 |
| CoxBoost+GBM | FUS |
| CoxBoost+GBM | HES4 |
| CoxBoost+GBM | PTTG1 |
| CoxBoost+GBM | SNRNP70 |
| CoxBoost+Lasso | CDC20 |
| CoxBoost+Lasso | FUS |
| CoxBoost+Lasso | HES4 |
| CoxBoost+Lasso | PTTG1 |
| CoxBoost+Lasso | SNRNP70 |
| CoxBoost+RSF | CDC20 |
| CoxBoost+RSF | FUS |
| CoxBoost+RSF | HES4 |
| CoxBoost+RSF | PTTG1 |
| CoxBoost+RSF | SNRNP70 |
| CoxBoost+Ridge | CDC20 |
| CoxBoost+Ridge | FUS |
| CoxBoost+Ridge | HES4 |
| CoxBoost+Ridge | PTTG1 |
| CoxBoost+Ridge | SNRNP70 |
| CoxBoost+StepCox[forward] | CDC20 |
| CoxBoost+StepCox[forward] | FUS |
| CoxBoost+StepCox[forward] | HES4 |
| CoxBoost+StepCox[forward] | PTTG1 |
| CoxBoost+StepCox[forward] | SNRNP70 |
| CoxBoost+SuperPC | CDC20 |
| CoxBoost+SuperPC | FUS |
| CoxBoost+SuperPC | HES4 |
| CoxBoost+SuperPC | PTTG1 |
| CoxBoost+SuperPC | SNRNP70 |
| CoxBoost+plsRcox | CDC20 |
| CoxBoost+plsRcox | FUS |
| CoxBoost+plsRcox | HES4 |
| CoxBoost+plsRcox | PTTG1 |
| CoxBoost+plsRcox | SNRNP70 |
| CoxBoost+survivalSVM | CDC20 |
| CoxBoost+survivalSVM | FUS |
| CoxBoost+survivalSVM | HES4 |
| CoxBoost+survivalSVM | PTTG1 |
| CoxBoost+survivalSVM | SNRNP70 |
| Enet[alpha=0.1] | BIRC5 |
| Enet[alpha=0.1] | CBX2 |
| Enet[alpha=0.1] | CDC20 |
| Enet[alpha=0.1] | CDKN3 |
| Enet[alpha=0.1] | DEPDC1 |
| Enet[alpha=0.1] | ENSA |
| Enet[alpha=0.1] | FUS |
| Enet[alpha=0.1] | HES4 |
| Enet[alpha=0.1] | HLA-DPA1 |
| Enet[alpha=0.1] | HOOK2 |
| Enet[alpha=0.1] | HS3ST1 |
| Enet[alpha=0.1] | MYLK |
| Enet[alpha=0.1] | PTTG1 |
| Enet[alpha=0.1] | SERHL2 |
| Enet[alpha=0.1] | SNRNP70 |
| Enet[alpha=0.1] | SYNPO2 |
| Enet[alpha=0.2] | BIRC5 |
| Enet[alpha=0.2] | CBX2 |
| Enet[alpha=0.2] | CDC20 |
| Enet[alpha=0.2] | CDKN3 |
| Enet[alpha=0.2] | DEPDC1 |
| Enet[alpha=0.2] | ENSA |
| Enet[alpha=0.2] | FUS |
| Enet[alpha=0.2] | HES4 |
| Enet[alpha=0.2] | HLA-DPA1 |
| Enet[alpha=0.2] | HOOK2 |
| Enet[alpha=0.2] | HS3ST1 |
| Enet[alpha=0.2] | PTTG1 |
| Enet[alpha=0.2] | SERHL2 |
| Enet[alpha=0.2] | SNRNP70 |
| Enet[alpha=0.2] | SYNPO2 |
| Enet[alpha=0.3] | CBX2 |
| Enet[alpha=0.3] | CDC20 |
| Enet[alpha=0.3] | CDKN3 |
| Enet[alpha=0.3] | DEPDC1 |
| Enet[alpha=0.3] | ENSA |
| Enet[alpha=0.3] | FUS |
| Enet[alpha=0.3] | HES4 |
| Enet[alpha=0.3] | HLA-DPA1 |
| Enet[alpha=0.3] | HS3ST1 |
| Enet[alpha=0.3] | MYLK |
| Enet[alpha=0.3] | PTTG1 |
| Enet[alpha=0.3] | SERHL2 |
| Enet[alpha=0.3] | SNRNP70 |
| Enet[alpha=0.3] | SYNPO2 |
| Enet[alpha=0.4] | CBX2 |
| Enet[alpha=0.4] | CDC20 |
| Enet[alpha=0.4] | CDKN3 |
| Enet[alpha=0.4] | DEPDC1 |
| Enet[alpha=0.4] | ENSA |
| Enet[alpha=0.4] | FUS |
| Enet[alpha=0.4] | HES4 |
| Enet[alpha=0.4] | HLA-DPA1 |
| Enet[alpha=0.4] | HS3ST1 |
| Enet[alpha=0.4] | PTTG1 |
| Enet[alpha=0.4] | SNRNP70 |
| Enet[alpha=0.5] | CBX2 |
| Enet[alpha=0.5] | CDC20 |
| Enet[alpha=0.5] | DEPDC1 |
| Enet[alpha=0.5] | ENSA |
| Enet[alpha=0.5] | FUS |
| Enet[alpha=0.5] | HES4 |
| Enet[alpha=0.5] | HLA-DPA1 |
| Enet[alpha=0.5] | HS3ST1 |
| Enet[alpha=0.5] | PTTG1 |
| Enet[alpha=0.5] | SERHL2 |
| Enet[alpha=0.5] | SNRNP70 |
| Enet[alpha=0.6] | CDC20 |
| Enet[alpha=0.6] | FUS |
| Enet[alpha=0.6] | HES4 |
| Enet[alpha=0.6] | PTTG1 |
| Enet[alpha=0.6] | SNRNP70 |
| Enet[alpha=0.7] | CDC20 |
| Enet[alpha=0.7] | FUS |
| Enet[alpha=0.7] | HES4 |
| Enet[alpha=0.7] | PTTG1 |
| Enet[alpha=0.7] | SNRNP70 |
| Enet[alpha=0.8] | CDC20 |
| Enet[alpha=0.8] | FUS |
| Enet[alpha=0.8] | HES4 |
| Enet[alpha=0.8] | PTTG1 |
| Enet[alpha=0.8] | SNRNP70 |
| Enet[alpha=0.9] | CDC20 |
| Enet[alpha=0.9] | DEPDC1 |
| Enet[alpha=0.9] | FUS |
| Enet[alpha=0.9] | HES4 |
| Enet[alpha=0.9] | HLA-DPA1 |
| Enet[alpha=0.9] | HS3ST1 |
| Enet[alpha=0.9] | MYLK |
| Enet[alpha=0.9] | PTTG1 |
| Enet[alpha=0.9] | SERHL2 |
| Enet[alpha=0.9] | SNRNP70 |
| GBM | BIRC5 |
| GBM | CBX2 |
| GBM | CDC20 |
| GBM | CDKN3 |
| GBM | DEPDC1 |
| GBM | ENSA |
| GBM | FUS |
| GBM | HES4 |
| GBM | HOOK2 |
| GBM | HPSE2 |
| GBM | HS3ST1 |
| GBM | MYLK |
| GBM | NRP1 |
| GBM | OGN |
| GBM | PTTG1 |
| GBM | SERHL2 |
| GBM | SNRNP70 |
| GBM | SYNPO2 |
| GBM | TIMM44 |
| GBM | TOP2A |
| GBM | `HLA-DPA1` |
| Lasso | CDC20 |
| Lasso | FUS |
| Lasso | HES4 |
| Lasso | PTTG1 |
| Lasso | SNRNP70 |
| Lasso+CoxBoost | CDC20 |
| Lasso+CoxBoost | FUS |
| Lasso+CoxBoost | HES4 |
| Lasso+CoxBoost | PTTG1 |
| Lasso+CoxBoost | SNRNP70 |
| Lasso+GBM | CDC20 |
| Lasso+GBM | FUS |
| Lasso+GBM | HES4 |
| Lasso+GBM | PTTG1 |
| Lasso+GBM | SNRNP70 |
| Lasso+RSF | CDC20 |
| Lasso+RSF | FUS |
| Lasso+RSF | HES4 |
| Lasso+RSF | PTTG1 |
| Lasso+RSF | SNRNP70 |
| Lasso+StepCox[forward] | CDC20 |
| Lasso+StepCox[forward] | FUS |
| Lasso+StepCox[forward] | HES4 |
| Lasso+StepCox[forward] | PTTG1 |
| Lasso+StepCox[forward] | SNRNP70 |
| Lasso+SuperPC | CDC20 |
| Lasso+SuperPC | FUS |
| Lasso+SuperPC | HES4 |
| Lasso+SuperPC | PTTG1 |
| Lasso+SuperPC | SNRNP70 |
| Lasso+plsRcox | CDC20 |
| Lasso+plsRcox | FUS |
| Lasso+plsRcox | HES4 |
| Lasso+plsRcox | PTTG1 |
| Lasso+plsRcox | SNRNP70 |
| Lasso+survivalSVM | CDC20 |
| Lasso+survivalSVM | FUS |
| Lasso+survivalSVM | HES4 |
| Lasso+survivalSVM | PTTG1 |
| Lasso+survivalSVM | SNRNP70 |
| RSF | CBX2 |
| RSF | CDC20 |
| RSF | CDKN3 |
| RSF | DEPDC1 |
| RSF | ENSA |
| RSF | FUS |
| RSF | HS3ST1 |
| RSF | PTTG1 |
| RSF | SERHL2 |
| RSF | SNRNP70 |
| RSF | TIMM44 |
| RSF+CoxBoost | CDC20 |
| RSF+CoxBoost | FUS |
| RSF+CoxBoost | PTTG1 |
| RSF+CoxBoost | SNRNP70 |
| RSF+Enet[alpha=0.1] | CBX2 |
| RSF+Enet[alpha=0.1] | CDC20 |
| RSF+Enet[alpha=0.1] | CDKN3 |
| RSF+Enet[alpha=0.1] | DEPDC1 |
| RSF+Enet[alpha=0.1] | ENSA |
| RSF+Enet[alpha=0.1] | FUS |
| RSF+Enet[alpha=0.1] | HOOK2 |
| RSF+Enet[alpha=0.1] | HS3ST1 |
| RSF+Enet[alpha=0.1] | PTTG1 |
| RSF+Enet[alpha=0.1] | SERHL2 |
| RSF+Enet[alpha=0.1] | SNRNP70 |
| RSF+Enet[alpha=0.2] | CBX2 |
| RSF+Enet[alpha=0.2] | CDC20 |
| RSF+Enet[alpha=0.2] | CDKN3 |
| RSF+Enet[alpha=0.2] | DEPDC1 |
| RSF+Enet[alpha=0.2] | ENSA |
| RSF+Enet[alpha=0.2] | FUS |
| RSF+Enet[alpha=0.2] | HOOK2 |
| RSF+Enet[alpha=0.2] | HS3ST1 |
| RSF+Enet[alpha=0.2] | PTTG1 |
| RSF+Enet[alpha=0.2] | SERHL2 |
| RSF+Enet[alpha=0.2] | SNRNP70 |
| RSF+Enet[alpha=0.3] | CBX2 |
| RSF+Enet[alpha=0.3] | CDC20 |
| RSF+Enet[alpha=0.3] | CDKN3 |
| RSF+Enet[alpha=0.3] | DEPDC1 |
| RSF+Enet[alpha=0.3] | ENSA |
| RSF+Enet[alpha=0.3] | FUS |
| RSF+Enet[alpha=0.3] | HOOK2 |
| RSF+Enet[alpha=0.3] | HS3ST1 |
| RSF+Enet[alpha=0.3] | PTTG1 |
| RSF+Enet[alpha=0.3] | SERHL2 |
| RSF+Enet[alpha=0.3] | SNRNP70 |
| RSF+Enet[alpha=0.4] | CBX2 |
| RSF+Enet[alpha=0.4] | CDC20 |
| RSF+Enet[alpha=0.4] | DEPDC1 |
| RSF+Enet[alpha=0.4] | ENSA |
| RSF+Enet[alpha=0.4] | FUS |
| RSF+Enet[alpha=0.4] | HS3ST1 |
| RSF+Enet[alpha=0.4] | PTTG1 |
| RSF+Enet[alpha=0.4] | SERHL2 |
| RSF+Enet[alpha=0.4] | SNRNP70 |
| RSF+Enet[alpha=0.5] | CBX2 |
| RSF+Enet[alpha=0.5] | CDC20 |
| RSF+Enet[alpha=0.5] | DEPDC1 |
| RSF+Enet[alpha=0.5] | ENSA |
| RSF+Enet[alpha=0.5] | FUS |
| RSF+Enet[alpha=0.5] | HOOK2 |
| RSF+Enet[alpha=0.5] | HS3ST1 |
| RSF+Enet[alpha=0.5] | PTTG1 |
| RSF+Enet[alpha=0.5] | SERHL2 |
| RSF+Enet[alpha=0.5] | SNRNP70 |
| RSF+Enet[alpha=0.6] | CBX2 |
| RSF+Enet[alpha=0.6] | CDC20 |
| RSF+Enet[alpha=0.6] | DEPDC1 |
| RSF+Enet[alpha=0.6] | ENSA |
| RSF+Enet[alpha=0.6] | FUS |
| RSF+Enet[alpha=0.6] | HS3ST1 |
| RSF+Enet[alpha=0.6] | PTTG1 |
| RSF+Enet[alpha=0.6] | SERHL2 |
| RSF+Enet[alpha=0.6] | SNRNP70 |
| RSF+Enet[alpha=0.7] | CBX2 |
| RSF+Enet[alpha=0.7] | CDC20 |
| RSF+Enet[alpha=0.7] | DEPDC1 |
| RSF+Enet[alpha=0.7] | FUS |
| RSF+Enet[alpha=0.7] | HS3ST1 |
| RSF+Enet[alpha=0.7] | PTTG1 |
| RSF+Enet[alpha=0.7] | SNRNP70 |
| RSF+Enet[alpha=0.8] | CDC20 |
| RSF+Enet[alpha=0.8] | FUS |
| RSF+Enet[alpha=0.8] | PTTG1 |
| RSF+Enet[alpha=0.8] | SNRNP70 |
| RSF+Enet[alpha=0.9] | CBX2 |
| RSF+Enet[alpha=0.9] | CDC20 |
| RSF+Enet[alpha=0.9] | DEPDC1 |
| RSF+Enet[alpha=0.9] | FUS |
| RSF+Enet[alpha=0.9] | PTTG1 |
| RSF+Enet[alpha=0.9] | SNRNP70 |
| RSF+GBM | CBX2 |
| RSF+GBM | CDC20 |
| RSF+GBM | CDKN3 |
| RSF+GBM | DEPDC1 |
| RSF+GBM | ENSA |
| RSF+GBM | FUS |
| RSF+GBM | HOOK2 |
| RSF+GBM | HS3ST1 |
| RSF+GBM | PTTG1 |
| RSF+GBM | SERHL2 |
| RSF+GBM | SNRNP70 |
| RSF+GBM | TIMM44 |
| RSF+Lasso | CDC20 |
| RSF+Lasso | FUS |
| RSF+Lasso | PTTG1 |
| RSF+Lasso | SNRNP70 |
| RSF+Ridge | CBX2 |
| RSF+Ridge | CDC20 |
| RSF+Ridge | CDKN3 |
| RSF+Ridge | DEPDC1 |
| RSF+Ridge | ENSA |
| RSF+Ridge | FUS |
| RSF+Ridge | HOOK2 |
| RSF+Ridge | HS3ST1 |
| RSF+Ridge | PTTG1 |
| RSF+Ridge | SERHL2 |
| RSF+Ridge | SNRNP70 |
| RSF+Ridge | TIMM44 |
| RSF+StepCox[backward] | DEPDC1 |
| RSF+StepCox[backward] | FUS |
| RSF+StepCox[backward] | PTTG1 |
| RSF+StepCox[backward] | SNRNP70 |
| RSF+StepCox[both] | DEPDC1 |
| RSF+StepCox[both] | FUS |
| RSF+StepCox[both] | PTTG1 |
| RSF+StepCox[both] | SNRNP70 |
| RSF+StepCox[forward] | CBX2 |
| RSF+StepCox[forward] | CDC20 |
| RSF+StepCox[forward] | CDKN3 |
| RSF+StepCox[forward] | DEPDC1 |
| RSF+StepCox[forward] | ENSA |
| RSF+StepCox[forward] | FUS |
| RSF+StepCox[forward] | HOOK2 |
| RSF+StepCox[forward] | HS3ST1 |
| RSF+StepCox[forward] | PTTG1 |
| RSF+StepCox[forward] | SERHL2 |
| RSF+StepCox[forward] | SNRNP70 |
| RSF+StepCox[forward] | TIMM44 |
| RSF+plsRcox | CBX2 |
| RSF+plsRcox | CDC20 |
| RSF+plsRcox | CDKN3 |
| RSF+plsRcox | DEPDC1 |
| RSF+plsRcox | ENSA |
| RSF+plsRcox | FUS |
| RSF+plsRcox | HOOK2 |
| RSF+plsRcox | HS3ST1 |
| RSF+plsRcox | PTTG1 |
| RSF+plsRcox | SERHL2 |
| RSF+plsRcox | SNRNP70 |
| RSF+plsRcox | TIMM44 |
| RSF+survivalSVM | CBX2 |
| RSF+survivalSVM | CDC20 |
| RSF+survivalSVM | CDKN3 |
| RSF+survivalSVM | DEPDC1 |
| RSF+survivalSVM | ENSA |
| RSF+survivalSVM | FUS |
| RSF+survivalSVM | HOOK2 |
| RSF+survivalSVM | HS3ST1 |
| RSF+survivalSVM | PTTG1 |
| RSF+survivalSVM | SERHL2 |
| RSF+survivalSVM | SNRNP70 |
| RSF+survivalSVM | TIMM44 |
| Ridge | BIRC5 |
| Ridge | CBX2 |
| Ridge | CDC20 |
| Ridge | CDKN3 |
| Ridge | DEPDC1 |
| Ridge | ENSA |
| Ridge | FUS |
| Ridge | HES4 |
| Ridge | HLA-DPA1 |
| Ridge | HOOK2 |
| Ridge | HPSE2 |
| Ridge | HS3ST1 |
| Ridge | MYLK |
| Ridge | NRP1 |
| Ridge | OGN |
| Ridge | PTTG1 |
| Ridge | SERHL2 |
| Ridge | SNRNP70 |
| Ridge | SYNPO2 |
| Ridge | TIMM44 |
| Ridge | TOP2A |
| StepCox[backward] | CDC20 |
| StepCox[backward] | DEPDC1 |
| StepCox[backward] | FUS |
| StepCox[backward] | HLA-DPA1 |
| StepCox[backward] | TOP2A |
| StepCox[backward]+CoxBoost | CDC20 |
| StepCox[backward]+CoxBoost | DEPDC1 |
| StepCox[backward]+CoxBoost | FUS |
| StepCox[backward]+CoxBoost | HLA-DPA1 |
| StepCox[backward]+CoxBoost | TOP2A |
| StepCox[backward]+Enet[alpha=0.1] | CDC20 |
| StepCox[backward]+Enet[alpha=0.1] | DEPDC1 |
| StepCox[backward]+Enet[alpha=0.1] | FUS |
| StepCox[backward]+Enet[alpha=0.1] | HLA-DPA1 |
| StepCox[backward]+Enet[alpha=0.1] | TOP2A |
| StepCox[backward]+Enet[alpha=0.2] | CDC20 |
| StepCox[backward]+Enet[alpha=0.2] | DEPDC1 |
| StepCox[backward]+Enet[alpha=0.2] | FUS |
| StepCox[backward]+Enet[alpha=0.2] | HLA-DPA1 |
| StepCox[backward]+Enet[alpha=0.2] | TOP2A |
| StepCox[backward]+Enet[alpha=0.3] | CDC20 |
| StepCox[backward]+Enet[alpha=0.3] | DEPDC1 |
| StepCox[backward]+Enet[alpha=0.3] | FUS |
| StepCox[backward]+Enet[alpha=0.3] | HLA-DPA1 |
| StepCox[backward]+Enet[alpha=0.3] | TOP2A |
| StepCox[backward]+Enet[alpha=0.4] | CDC20 |
| StepCox[backward]+Enet[alpha=0.4] | DEPDC1 |
| StepCox[backward]+Enet[alpha=0.4] | FUS |
| StepCox[backward]+Enet[alpha=0.4] | HLA-DPA1 |
| StepCox[backward]+Enet[alpha=0.4] | TOP2A |
| StepCox[backward]+Enet[alpha=0.5] | CDC20 |
| StepCox[backward]+Enet[alpha=0.5] | DEPDC1 |
| StepCox[backward]+Enet[alpha=0.5] | FUS |
| StepCox[backward]+Enet[alpha=0.5] | HLA-DPA1 |
| StepCox[backward]+Enet[alpha=0.5] | TOP2A |
| StepCox[backward]+Enet[alpha=0.6] | CDC20 |
| StepCox[backward]+Enet[alpha=0.6] | DEPDC1 |
| StepCox[backward]+Enet[alpha=0.6] | FUS |
| StepCox[backward]+Enet[alpha=0.6] | HLA-DPA1 |
| StepCox[backward]+Enet[alpha=0.6] | TOP2A |
| StepCox[backward]+Enet[alpha=0.7] | CDC20 |
| StepCox[backward]+Enet[alpha=0.7] | DEPDC1 |
| StepCox[backward]+Enet[alpha=0.7] | FUS |
| StepCox[backward]+Enet[alpha=0.7] | HLA-DPA1 |
| StepCox[backward]+Enet[alpha=0.7] | TOP2A |
| StepCox[backward]+Enet[alpha=0.8] | CDC20 |
| StepCox[backward]+Enet[alpha=0.8] | DEPDC1 |
| StepCox[backward]+Enet[alpha=0.8] | FUS |
| StepCox[backward]+Enet[alpha=0.8] | HLA-DPA1 |
| StepCox[backward]+Enet[alpha=0.8] | TOP2A |
| StepCox[backward]+Enet[alpha=0.9] | CDC20 |
| StepCox[backward]+Enet[alpha=0.9] | DEPDC1 |
| StepCox[backward]+Enet[alpha=0.9] | FUS |
| StepCox[backward]+Enet[alpha=0.9] | HLA-DPA1 |
| StepCox[backward]+Enet[alpha=0.9] | TOP2A |
| StepCox[backward]+GBM | CDC20 |
| StepCox[backward]+GBM | DEPDC1 |
| StepCox[backward]+GBM | FUS |
| StepCox[backward]+GBM | TOP2A |
| StepCox[backward]+GBM | `HLA-DPA1` |
| StepCox[backward]+Lasso | CDC20 |
| StepCox[backward]+Lasso | DEPDC1 |
| StepCox[backward]+Lasso | FUS |
| StepCox[backward]+Lasso | HLA-DPA1 |
| StepCox[backward]+Lasso | TOP2A |
| StepCox[backward]+RSF | CDC20 |
| StepCox[backward]+RSF | DEPDC1 |
| StepCox[backward]+RSF | FUS |
| StepCox[backward]+RSF | HLA-DPA1 |
| StepCox[backward]+RSF | TOP2A |
| StepCox[backward]+Ridge | CDC20 |
| StepCox[backward]+Ridge | DEPDC1 |
| StepCox[backward]+Ridge | FUS |
| StepCox[backward]+Ridge | HLA-DPA1 |
| StepCox[backward]+Ridge | TOP2A |
| StepCox[backward]+SuperPC | CDC20 |
| StepCox[backward]+SuperPC | DEPDC1 |
| StepCox[backward]+SuperPC | FUS |
| StepCox[backward]+SuperPC | HLA-DPA1 |
| StepCox[backward]+SuperPC | TOP2A |
| StepCox[backward]+plsRcox | CDC20 |
| StepCox[backward]+plsRcox | DEPDC1 |
| StepCox[backward]+plsRcox | FUS |
| StepCox[backward]+plsRcox | HLA.DPA1 |
| StepCox[backward]+plsRcox | TOP2A |
| StepCox[backward]+survivalSVM | CDC20 |
| StepCox[backward]+survivalSVM | DEPDC1 |
| StepCox[backward]+survivalSVM | FUS |
| StepCox[backward]+survivalSVM | HLA-DPA1 |
| StepCox[backward]+survivalSVM | TOP2A |
| StepCox[both] | CDC20 |
| StepCox[both] | DEPDC1 |
| StepCox[both] | FUS |
| StepCox[both] | HLA-DPA1 |
| StepCox[both] | TOP2A |
| StepCox[both]+CoxBoost | CDC20 |
| StepCox[both]+CoxBoost | DEPDC1 |
| StepCox[both]+CoxBoost | FUS |
| StepCox[both]+CoxBoost | HLA-DPA1 |
| StepCox[both]+CoxBoost | TOP2A |
| StepCox[both]+Enet[alpha=0.1] | CDC20 |
| StepCox[both]+Enet[alpha=0.1] | DEPDC1 |
| StepCox[both]+Enet[alpha=0.1] | FUS |
| StepCox[both]+Enet[alpha=0.1] | HLA-DPA1 |
| StepCox[both]+Enet[alpha=0.1] | TOP2A |
| StepCox[both]+Enet[alpha=0.2] | CDC20 |
| StepCox[both]+Enet[alpha=0.2] | DEPDC1 |
| StepCox[both]+Enet[alpha=0.2] | FUS |
| StepCox[both]+Enet[alpha=0.2] | HLA-DPA1 |
| StepCox[both]+Enet[alpha=0.2] | TOP2A |
| StepCox[both]+Enet[alpha=0.3] | CDC20 |
| StepCox[both]+Enet[alpha=0.3] | DEPDC1 |
| StepCox[both]+Enet[alpha=0.3] | FUS |
| StepCox[both]+Enet[alpha=0.3] | HLA-DPA1 |
| StepCox[both]+Enet[alpha=0.3] | TOP2A |
| StepCox[both]+Enet[alpha=0.4] | CDC20 |
| StepCox[both]+Enet[alpha=0.4] | DEPDC1 |
| StepCox[both]+Enet[alpha=0.4] | FUS |
| StepCox[both]+Enet[alpha=0.4] | HLA-DPA1 |
| StepCox[both]+Enet[alpha=0.4] | TOP2A |
| StepCox[both]+Enet[alpha=0.5] | CDC20 |
| StepCox[both]+Enet[alpha=0.5] | DEPDC1 |
| StepCox[both]+Enet[alpha=0.5] | FUS |
| StepCox[both]+Enet[alpha=0.5] | HLA-DPA1 |
| StepCox[both]+Enet[alpha=0.5] | TOP2A |
| StepCox[both]+Enet[alpha=0.6] | CDC20 |
| StepCox[both]+Enet[alpha=0.6] | DEPDC1 |
| StepCox[both]+Enet[alpha=0.6] | FUS |
| StepCox[both]+Enet[alpha=0.6] | HLA-DPA1 |
| StepCox[both]+Enet[alpha=0.6] | TOP2A |
| StepCox[both]+Enet[alpha=0.7] | CDC20 |
| StepCox[both]+Enet[alpha=0.7] | DEPDC1 |
| StepCox[both]+Enet[alpha=0.7] | FUS |
| StepCox[both]+Enet[alpha=0.7] | HLA-DPA1 |
| StepCox[both]+Enet[alpha=0.7] | TOP2A |
| StepCox[both]+Enet[alpha=0.8] | CDC20 |
| StepCox[both]+Enet[alpha=0.8] | DEPDC1 |
| StepCox[both]+Enet[alpha=0.8] | FUS |
| StepCox[both]+Enet[alpha=0.8] | HLA-DPA1 |
| StepCox[both]+Enet[alpha=0.8] | TOP2A |
| StepCox[both]+Enet[alpha=0.9] | CDC20 |
| StepCox[both]+Enet[alpha=0.9] | DEPDC1 |
| StepCox[both]+Enet[alpha=0.9] | FUS |
| StepCox[both]+Enet[alpha=0.9] | HLA-DPA1 |
| StepCox[both]+Enet[alpha=0.9] | TOP2A |
| StepCox[both]+GBM | CDC20 |
| StepCox[both]+GBM | DEPDC1 |
| StepCox[both]+GBM | FUS |
| StepCox[both]+GBM | TOP2A |
| StepCox[both]+GBM | `HLA-DPA1` |
| StepCox[both]+Lasso | CDC20 |
| StepCox[both]+Lasso | DEPDC1 |
| StepCox[both]+Lasso | FUS |
| StepCox[both]+Lasso | HLA-DPA1 |
| StepCox[both]+Lasso | TOP2A |
| StepCox[both]+RSF | CDC20 |
| StepCox[both]+RSF | DEPDC1 |
| StepCox[both]+RSF | FUS |
| StepCox[both]+RSF | HLA-DPA1 |
| StepCox[both]+RSF | TOP2A |
| StepCox[both]+Ridge | CDC20 |
| StepCox[both]+Ridge | DEPDC1 |
| StepCox[both]+Ridge | FUS |
| StepCox[both]+Ridge | HLA-DPA1 |
| StepCox[both]+Ridge | TOP2A |
| StepCox[both]+SuperPC | CDC20 |
| StepCox[both]+SuperPC | DEPDC1 |
| StepCox[both]+SuperPC | FUS |
| StepCox[both]+SuperPC | HLA-DPA1 |
| StepCox[both]+SuperPC | TOP2A |
| StepCox[both]+plsRcox | CDC20 |
| StepCox[both]+plsRcox | DEPDC1 |
| StepCox[both]+plsRcox | FUS |
| StepCox[both]+plsRcox | HLA.DPA1 |
| StepCox[both]+plsRcox | TOP2A |
| StepCox[both]+survivalSVM | CDC20 |
| StepCox[both]+survivalSVM | DEPDC1 |
| StepCox[both]+survivalSVM | FUS |
| StepCox[both]+survivalSVM | HLA-DPA1 |
| StepCox[both]+survivalSVM | TOP2A |
| StepCox[forward] | BIRC5 |
| StepCox[forward] | CBX2 |
| StepCox[forward] | CDC20 |
| StepCox[forward] | CDKN3 |
| StepCox[forward] | DEPDC1 |
| StepCox[forward] | ENSA |
| StepCox[forward] | FUS |
| StepCox[forward] | HES4 |
| StepCox[forward] | HLA-DPA1 |
| StepCox[forward] | HOOK2 |
| StepCox[forward] | HPSE2 |
| StepCox[forward] | HS3ST1 |
| StepCox[forward] | MYLK |
| StepCox[forward] | NRP1 |
| StepCox[forward] | OGN |
| StepCox[forward] | PTTG1 |
| StepCox[forward] | SERHL2 |
| StepCox[forward] | SNRNP70 |
| StepCox[forward] | SYNPO2 |
| StepCox[forward] | TIMM44 |
| StepCox[forward] | TOP2A |
| SuperPC | BIRC5 |
| SuperPC | CBX2 |
| SuperPC | CDC20 |
| SuperPC | CDKN3 |
| SuperPC | DEPDC1 |
| SuperPC | ENSA |
| SuperPC | FUS |
| SuperPC | HES4 |
| SuperPC | HLA-DPA1 |
| SuperPC | HOOK2 |
| SuperPC | HPSE2 |
| SuperPC | HS3ST1 |
| SuperPC | MYLK |
| SuperPC | NRP1 |
| SuperPC | OGN |
| SuperPC | PTTG1 |
| SuperPC | SERHL2 |
| SuperPC | SNRNP70 |
| SuperPC | SYNPO2 |
| SuperPC | TIMM44 |
| SuperPC | TOP2A |
| plsRcox | BIRC5 |
| plsRcox | CBX2 |
| plsRcox | CDC20 |
| plsRcox | CDKN3 |
| plsRcox | DEPDC1 |
| plsRcox | ENSA |
| plsRcox | FUS |
| plsRcox | HES4 |
| plsRcox | HLA.DPA1 |
| plsRcox | HOOK2 |
| plsRcox | HPSE2 |
| plsRcox | HS3ST1 |
| plsRcox | MYLK |
| plsRcox | NRP1 |
| plsRcox | OGN |
| plsRcox | PTTG1 |
| plsRcox | SERHL2 |
| plsRcox | SNRNP70 |
| plsRcox | SYNPO2 |
| plsRcox | TIMM44 |
| plsRcox | TOP2A |
| survivalSVM | BIRC5 |
| survivalSVM | CBX2 |
| survivalSVM | CDC20 |
| survivalSVM | CDKN3 |
| survivalSVM | DEPDC1 |
| survivalSVM | ENSA |
| survivalSVM | FUS |
| survivalSVM | HES4 |
| survivalSVM | HLA-DPA1 |
| survivalSVM | HOOK2 |
| survivalSVM | HPSE2 |
| survivalSVM | HS3ST1 |
| survivalSVM | MYLK |
| survivalSVM | NRP1 |
| survivalSVM | OGN |
| survivalSVM | PTTG1 |
| survivalSVM | SERHL2 |
| survivalSVM | SNRNP70 |
| survivalSVM | SYNPO2 |
| survivalSVM | TIMM44 |
| survivalSVM | TOP2A |
| nan | HOOK2 RSF |

## Supplementary Figure S1. Distribution of Scissor classes across patients

Stacked barplot showing the fraction of Scissor+, Scissor−, and Background cells among malignant luminal cells for each of the 13 patients (P53–P77) in the GSE141445 single-cell dataset. Both Scissor+ and Scissor− cells were represented across multiple patients, with varying relative proportions between patients.
